# Quality evaluation of oil by cold‐pressed peanut from different growing regions in China

**DOI:** 10.1002/fsn3.2813

**Published:** 2022-03-14

**Authors:** Ying Huang, Changsheng Liu, Fenghong Huang, Qi Zhou, Chang Zheng, Rui Liu, Jiazhang Huang

**Affiliations:** ^1^ Oil Crops Research Institute Chinese Academy of Agricultural Sciences Key Laboratory of Oilseeds Processing Ministry of Agriculture and Rural Affairs of the People’s Republic of China Oil Crops and Lipids Process Technology National & Local Joint Engineering Laboratory, Hubei Key Laboratory of Lipid Chemistry and Nutrition Wuhan China; ^2^ Institute of Food and Nutrition Development Ministry of Agriculture and Rural Affairs Beijing China

**Keywords:** different varieties, fatty acids, growing region, nutritional characteristic, PCA, peanut oil

## Abstract

In this study, twenty‐six peanut varieties and their cold‐pressed oils from eleven provinces in China were investigated for their oil content, acid value, peroxide value, fatty acid profiles, bioactive constituents, and induction period (IP) of lipid oxidation. Meanwhile, the effect of the geographical origin of peanut on the quality of cold‐pressed peanut oils (CPOs) was studied. The average acid value of CPOs in southern China was higher than that in northern China (0.49 mg KOH/g versus 0.22 mg KOH/g, *p >* .05). In addition, the average of oleic acid content, ratio of oleic acid to linoleic acid (O/L), and IP were also higher in southern China than that in northern China (*p <* .05). However, the average content of campesterol, *β*‐sitosterol, total phytosterol, linoleic acid, and ratio of unsaturated fatty acid to saturated fatty acid (UFA/SFA) exhibited reverse results (*p* < .05). At last, the comprehensive evaluation of CPOs based on principal component analysis (PCA) was performed. In all samples, Silihong from Liaoning province, northern China was No.1, and Zhonghua 21 from Xiaogan City, Hubei Province was No.4 which was the first one from southern China. Moreover, heat map clustering analysis further revealed the differences and similarities among different samples, and those results were in accordance with the comprehensive evaluation results.

## Graphical abstract

The physicochemical and nutritional properties of cold pressed oil from 26 representative varieties in China show significant difference. The PCA and cluster analysis suggest that the top rankings of peanut oil quality are mostly in the north.

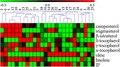

## 1 INTRODUCTION

Peanut (*Arachis hypogaea L*.) is an economically important oil seed crop grown in tropical and subtropical agro‐climatic areas of Asia, Africa, and the Americas with the oil content in the range of 44%–56% (Verma et al., 2012). China, India, and the United States are the top three peanut producers in the world. By the end of 2020, the global peanut production reached 50.53 million metric tons, of which 36.02% was provided by China. Meanwhile, China is also a major consumer of peanut oil, consuming around 3.54 million metric tons in 2020–2021, accounting for 54.81% of the world's total domestic consumption (USDA, 2021).

Peanut oil is a rich source of dietary essential fatty acids including oleic acids (36% to 67%) and linoleic acids (15% to 46%), and O/L ratios are in the range of 1.19–4.46 (Akram et al., 2018). The previous studies have shown that O/L is associated with a lower risk of cardiovascular disease, and linoleic acid has a positive influence on coronary heart disease (Dun et al., 2019). Furthermore, it contains several biologically active ingredients for instance tocopherols and phytosterols. *A*‐tocopherol and *γ*‐tocopherol are the major form of vitamin E in peanut oil, which has good lipid antioxidant capacity (Carrín & Carelli, 2010). Phytosterols can retard the development of atherosclerosis, lower the risk of type 2 diabetes, and reduce the risk of colorectal cancer, which is conducive to human health benefits (Suchoszek‐Łukaniuk et al., 2011).

At present, studies tend to concentrate on the effect of different processing technologies on the chemical components and quality changes in peanut oil. However, different planting areas may lead to quality variation in peanut resources, which in turn leads to change in processed quality. Sanders et al. (1992) found that tocopherol content was obviously different in peanuts from various regions. The fatty acid composition and oil stability was variable depending on the growth location and cultivar (Campos‐Mondragón et al., 2009; Grosso & Guzman, 1995; Grosso et al., 1994). Although several researchers have confirmed the view that natural conditions played a significant role in oil stability and quality, differences in Chinese peanut varieties, especially from the north and south regions, have not been extensively studied. The south and the north have been the largest symbolic geographical division and two major peanut production areas since ancient times, which show a completely different ecological environment, reflected in climate, rainfall, soil, and sunlight.

The purpose of this research was to capture a more detailed understanding of the processing characteristics of peanut from different regions and varieties by evaluating the effect of different planting areas (north–south region) on the physicochemical and nutritional characteristics of cold‐pressed peanut oil, thereby providing scientific guidance for processing high‐quality peanut oil and breeding peanut varieties.

## 2 MATERIALS AND METHODS

### 2.1 Materials and chemicals

Twenty‐six peanut cultivars (*Arachis hypogaea L*.) planted from the Chinese main planting areas in September (October) 2019 were collected, cleaned, and dried at room temperature. With Qinling and Huaihe as dividing lines, fourteen kinds came from the southern region, including Sichuan (one cultivars), Guangxi (two cultivars), Guangdong (one cultivar), Jiangsu (one cultivar), Jiangxi (three cultivars), Hunan (two cultivars), and Hubei (four cultivars); twelve kinds came from the northern region, including Hebei (three cultivars), Henan (two cultivars), Shandong (four cultivars), Jiangsu (one cultivar) and Liaoning (two cultivars) regions. More details about peanut samples are listed in Table S1.

*α‐, γ‐,* and *δ‐*tocopherol (purity ≥98%), cholesterol (purity ≥99%), stigmasterol (purity ≥95%), *β*‐sitosterol (purity ≥95%) and campesterol (purity ≥98%), 5*α*‐cholestane (purity ≥97%), and N,O‐Bis (trimethylsilyl) trifluoroacetamide with trimethylchlorosilane (BSTFA+TMCS ≥ 98.5%, excluding trimethylchlorosilane) were purchased from Sigma‐Aldrich (Saint Louis, MS, USA). Chromatographic grade isopropanol and hexane were purchased from Merck (Darmstadt, Hesse, Germany). Other chemicals and reagents were of analytical grade and purchased from Chinese medicine group chemical reagent co., Ltd (Shanghai, China).

### 2.2 Cold‐pressed peanut oil extraction

All the above peanut seeds were pressed with a cold pressing machine (CA59G, German Monforts Group, Moenchen‐gladbach, North Rhine‐Westphalia, Germany) at a temperature below 60°C. Each oil obtained was centrifuged at 25,230 × g for 15 min (Avanti J‐26 XP, Beckman Coulter Inc., Brea, CA, USA) to remove residue and then kept cold (4 ± 2°C) for future experimental treatments.

### 2.3 Quality indices

The oil content in peanut seed was determined according to the GB 5009.6–2016 (CNIS, 2016), which involves the gravimetric analysis with analytical grade petroleum ether in a Soxhlet apparatus (B‐811; Buchi Labortechnik AG) for 8 hr. According to GB 5009.229–2016 and GB 5009.227–2016 (CNIS, 2016), the acid value and peroxide value are determined through titration method. The oil sample was fully dissolved in an organic solvent, and then titrated with KOH or sodium thiosulfate standard solution. Finally, the titration end point was defined by the color reaction of the indicator. The acid value was expressed as the milligrams of potassium hydroxide (KOH) required neutralizing 1 g of free fatty acid. The peroxide value was expressed as the millimoles of active oxygen in 1 kg of the sample.

### 2.4 Fatty acid profile

Preparation of fatty acid methyl ester (FAME) samples, reference to GB/T 5009.168–2016:60.0 mg (accurate to 0.1 mg) oil was taken into a 10‐mL centrifuge tube along with 4 ml isooctane and 200 μL of 2 mol/L methanol‐KOH solution. It was shaken for 30 s so that the sample mixed well, then set aside until clarified. Finally, 1 g of sodium bisulfate was fully used to neutralize potassium hydroxide. After the salt had precipitated, the upper phases were transferred to GC vials for testing.

Composition of fatty acids (%) was detected by gas chromatography (7890A, Agilent, U.S.A), as described by the European standard EN^14103^ (2003). 1 μL FAME specimen with a split ratio of 80:1 was injected into the capillary column (HP‐INNOWAX, 30 m × 0.32 mm ×0.25 μm; Agilent, Santa Clara, CA, USA) at a flow rate of 1.5 ml/min with nitrogen as the carrier gas. Oven temperature of the instrument increased from 210°C (kept for 9 min) to 230°C at a rate of 20°C /min and held for 10 min. The temperatures of the injector and FID detector were set to 250°C and 300°C. Chemstation software was used to calculate peak areas and retention time of individual fatty acid, and the content was expressed as the mass fractions.

### 2.5 Determination of tocopherols by high‐pressure liquid chromatography (HPLC)

The indigenous tocopherol contents of the twenty‐six peanut oils were determined by using AOCS official method Ce 8–89 *Tocopherols and Tocotrienols in Vegetable Oils and Fats by HPLC* (AOCS, 2009). In brief, the samples were accurately weighed at 2.0 g and dissolved in n‐hexane with a 25‐mL brown volumetric flask and made up to volume. It was important that the test solutions are protected from light prior to analysis and analyzed on the day of preparation. 20 µl of the test solution was injected onto the analytical column packed with micro particulate silica having a mean particle size of about 5 µm (250 × 4.8 mm) and tocopherols were detected by the diode array detector (SPDM20A, Shimadzu, Tokyo, Japan) at specific wavelength. The isocratic mobile phase was a mixture of n‐hexane: isopropanol (0.5:99.5, v/v) at a flow rate of 1.0 ml/min. Standards of *α‐, γ‐,* and *δ*‐tocopherol were diluted with hexane and their concentration was determined by the absorbance maximums of the solutions using UV spectroscopy according to Beer's Law (*λ* = 292 nm, 298 nm and 298 nm). Calculations of the unknown were done by comparison of peak areas and the calculated concentrations of the standard solutions. Standard curves of each isomer covered five orders of magnitude and bracketed all sample concentrations. Identify the tocopherols present by reference to the chromatograms obtained from standards and record the areas of the tocopherol peaks. The results were achieved through the formula conversion and expressed in milligram per kilogram (mg/kg).

### 2.6 GC analysis of the phytosterols

Phytosterols were measured according to the method described by Azadmard‐Damirchi et al. (2010), after minor modification. The weighed oil sample (*ca*.20 mg) was added to 0.5 ml of 5*α*‐cholesterol solution (0.5 mg/ml), then mixed thoroughly with 10 ml of 2 M KOH in 95% ethanol in a ground‐glass tube, and shaken in a water bath at 60°C for 60 min. After cooling, 4 ml of water and 10 ml of hexane were added and mixed vigorously. Thereafter, the mixture was centrifuged at 4863 g for 5 min and the hexane layer containing unsaponifiables was separated. The above process was carried out for three times, and the collected extract redissolved into 1ml hexane solution after complete evaporation, bottled for further analysis. Gas chromatographic conditions included a DB‐5HT column (30 m × 0.22 mm ×0.1 μm; Agilent, Santa Clara, CA, USA) performed at a flow rate of 1.5 ml/min with helium as the carrier gas. The injection volume was 1 μL, flow rate was 2 ml/min, and the split ratio was 25:1. The program temperature was maintained at 60°C for 1 min. Then, the temperature was increased at a rate of 40°C/min to a final temperature of 310°C, and held for 10 min.

According to the retention times of reference samples of phytosterols in the chromatogram, each phytosterol in the analyzed oil samples was identified. Quantification was done relative to the 5*α*‐cholestane as an internal standard. The calculation method was as follows:

$$X_{\delta }=\frac{0.25\times A_{\delta }\times 1000}{A_{\gamma }\times M}$$

where, X*_δ_* represents the content of a single sterol component, mg/kg; 0.25 represents the mass of 5*α*‐cholesterol, 0.5 mg / mL ×0.5 ml, mg; A*_δ_* represents a single sterol peak area; A*_γ_* represents 5*α*‐cholesterol peak area; M is the mass of the oil sample, g.

### 2.7 Oil stability

Oxidative stability was measured with the Rancimat 743 (Metrohm, Riverview, FL, USA), according to the method described by Yang et al. (2012). 3.0 g of oil samples were weighed into the reaction vessel in triplicate and heated to 110°C with an air flow of 20 L/h. Volatile products released during the oxidation process were collected in a flask containing distilled water. The oxidation process was recorded automatically by measuring the change in conductivity of the distilled water due to the formation of volatile compounds. The oxidative induction period (IP) was defined as the point of rapid change in the rate of oxidation, and the results were expressed in hours (h).

### 2.8 Statistical analysis and principal component analysis

The analysis was performed in triplicate. The obtained results were presented as the mean with standard deviation (*SD*). An independent sample *t*‐test (using SPSS 22 software; SPSS Inc., Chicago, IL, USA) was used to compare the mean value of two groups (CPOs in southern China versus. northern China). Mean differences were considered notably at the *p* <.05 level. The principal component analysis (PCA) and cluster analysis were performed for the nutritional comprehensive score of cold‐pressed peanut oils.

## 3 RESULTS AND DISCUSSION

### 3.1 Physicochemical characteristics

For such a large population like China, the development and utilization of edible oil is of vital importance. Peanuts can meet the oil intake needs of people and create a huge economic value. In addition, although peanuts are a high‐fat, energy‐dense food, clinical and epidemiological studies demonstrated that peanut consumption is not associated with weight gain (Mattes et al., 2008).

The oil content is an important feature of peanut seed evaluation, which may vary from 40% to 65% depending upon variety, season, and maturity. In this research, the influence of different provenances on the oil content of peanuts was emphasized, with the aim of providing reference for further selection of good seed materials with high oil content. On account of its guiding significance, the oil contents of twenty‐six peanut varieties ranged from 45.97% to 57.28% with statistical significance at the level of *p <*.05 (Table 1). The Luhua 9 (P10), coming from Xuzhou, Jiangsu, had the highest oil content (>57%). These values were in accordance with the report of Wang et al. (2012). In comparison to other typical oil crops, the peanut seeds contained a much higher proportion of oil than hemp seeds (26%–37%) and rapeseed (35%–39%) (Kriese et al., 2004; Yang et al., 2009).

**TABLE 1:** Physiochemical of CPOs from twenty‐six peanut cultivars grown in China

Sample	Oil content (g/100g)	Acid value (mg KOH/g)	Peroxide value (mmol/kg)
P1	48.58 ± 0.48^def^	0.13 ± 0.00^a^	3.70 ± 0.15^h^
P2	47.03 ± 0.16^b^	0.24 ± 0.01^efg^	3.01 ± 0.02^ef^
P3	49.55 ± 0.03^hij^	0.32 ± 0.00^k^	3.57 ± 0.04^h^
P4	49.72 ± 0.03^j^	0.24 ± 0.00^fgh^	5.55 ± 0.20^k^
P5	49.11 ± 0.01^fgh^	0.11 ± 0.00^a^	3.06 ± 0.44^ef^
P6	49.05 ± 0.12^fgh^	0.27 ± 0.00^j^	3.40 ± 0.17^h^
P7	49.14 ± 0.43^ghi^	0.24 ± 0.01^ghi^	3.12 ± 0.11^f^
P8	48.44 ± 0.21^de^	0.19 ± 0.00^c^	1.94 ± 0.16^a^
P9	49.06 ± 0.33^fgh^	0.30 ± 0.01^k^	3.62 ± 0.02^h^
P10	57.28 ± 0.01^o^	0.23 ± 0.01^fg^	2.16 ± 0.18^a^
P11	52.76 ± 0.11^m^	0.20 ± 0.01 cd	2.05 ± 0.11^a^
P12	52.71 ± 0.02^m^	0.17 ± 0.01^b^	2.20 ± 0.04^ab^
P13	45.97 ± 0.25^a^	0.22 ± 0.01^de^	4.91 ± 0.01^j^
P14	51.61 ± 0.32^l^	0.48 ± 0.01^o^	4.10 ± 0.28^i^
P15	49.66 ± 0.08^ij^	0.45 ± 0.00^n^	2.82 ± 0.10^cdef^
P16	51.43 ± 0.30^l^	0.58 ± 0.00^p^	2.63 ± 0.16 cd
P17	48.08 ± 0.38 cd	0.43 ± 0.01^m^	2.57 ± 0.01^c^
P18	48.80 ± 0.28^efg^	0.22 ± 0.00^f^	2.13 ± 0.13^a^
P19	46.59 ± 0.43^b^	0.36 ± 0.01^l^	2.71 ± 0.02 cd
P20	53.92 ± 0.26^n^	0.13 ± 0.01^a^	3.75 ± 0.34^h^
P21	50.69 ± 0.04^k^	0.26 ± 0.01^hij^	2.97 ± 0.04^cdef^
P22	50.62 ± 0.11^k^	0.16 ± 0.01^b^	2.05 ± 0.11^a^
P23	51.71 ± 0.01^l^	2.74 ± 0.04^q^	3.58 ± 0.10^h^
P24	45.97 ± 0.09^a^	0.36 ± 0.01^l^	4.39 ± 0.16^i^
P25	47.08 ± 0.27^b^	0.27 ± 0.01^ij^	2.53 ± 0.12^bc^
P26	47.80 ± 0.17^c^	0.23 ± 0.01^fg^	2.53 ± 0.03^bc^

Range and average value			
*N*	47.03–57.28 (50.20 ± 2.77^a^)	0.11–0.32 (0.22 ± 0.06^a^)	1.94–5.55 (3.11 ± 1.00^a^)
S	45.97–53.92 (49.28 ± 2.47^a^)	0.13–2.74 (0.49 ± 0.66^a^)	2.04–4.91 (3.12 ± 0.88^a^)

Note

*N* means peanuts grown in the north; S means peanuts grown in the south; values in the columns with different letters (a‐q) are significantly different (*p* <.05).

Physicochemical analyses represent parameters related to the conservation and quality of the oil, and is receiving more and more attention from producers, researchers, and consumers. These indexes indicate the conservation state of the oil based on the impact of the major environmental oxidants, such as heat, light, and oxygen, elements that can accelerate the decomposition of glycerides, develop rancidity, and lead to the formation of free fatty acids in the matrices (dos Santos et al., 2019). The acidity value, ranging from 0.11 mg KOH/g to 2.74 mg KOH/g obtained from twenty‐six samples (*p <*.05), was within the permitted level provided in GB 2716–2018 (≤3 mg KOH/g). The peroxide value (PV), which indicates the concentration of peroxides and hydroperoxides formed in the initial stages of lipid oxidation, can reflect the extent to which an oil is oxidized (Ni et al., 2015). In general, oils with peroxide value higher than 4.5 mmol/kg cause undesirable health problems by increasing reactive oxygen species as well as secondary products of lipid peroxidation that stimulate cardiovascular and inflammatory diseases (Konuskan et al., 2019). Oils with peroxide levels higher than 5 mmol/kg are considered to be less stable, and they have a short shelf life. In the test samples, Yuhua10 (P4) and Local red peanut (P13), with an initial peroxide value up to 5.55 mmol/kg and 4.91 mmol/kg, respectively, performed relatively poorly, which would quickly become unsuitable for human diets.

### 3.2 Identification and quantification of fatty acids

Oil composition is critical to final product quality of peanut‐based products, including nutritional profile, physical properties, flavor, and shelf life (Braddock et al., 2010). As basic constituents of fats and oils, fatty acids in different varieties of peanut oils were evaluated and identified (Table 2). The major fatty acids present were palmitic acid (C16:0), oleic acid (C18:1), and linoleic acid (C18:2). Additionally, palmitoleic acid (C16:1), stearic acids (C18:0), and longer chain fatty acids, such as arachidic acid (C20:0), gadoleic acid (C20:1), behenic acid (C22:0), lignoceric acid (C24:0), occurred in minor quantities. Across overall samples, relative (%) concentration of oleic acid revealed the highest amounts varied between 36.10% and 47.66%, followed by linoleic acid and palmitic acid in smaller amounts of 30.53%‐40.86% and 10.17%‐12.61%. The sum of these three fatty acids accounted for 88.46%‐90.60% of total fatty acids, which was consistent with published data by Wang (2018). Statistical analyses showed there existed observable differences (*p* <.01) among the peanut cultivars tested. Akhtar et al. (2014) concluded the fatty acid composition of peanut oil was highly variable according to the environmental conditions, variety, and peanut maturity level.

**TABLE 2:** Fatty acid composition and average content (%) of CPOs from twenty‐six peanut cultivars grown in China

Sample	Palmitic acid	Palmitoleic acid	Stearic acid	Oleic acid	Linoleic acids	Arachidic acid	Gadoleic acid	Behenic acid	Lignoceric acid	∑UFA	∑SFA	UFA/SFA	O/L
P1	12.04 ± 0.13^m^	0.26 ± 0.01 cd	3.38 ± 0.03^ef^	38.67 ± 0.16^d^	39.72 ± 0.10^p^	1.41 ± 0.01^ab^	0.77 ± 0.01^bcd^	2.43 ± 0.02^cde^	1.34 ± 0.15^c^	79.41 ± 0.27^jkl^	20.59 ± 0.27^def^	3.86 ± 0.06^ij^	0.97 ± 0.00^d^
P2	10.47 ± 0.06^b^	0.22 ± 0.01^bc^	2.83 ± 0.03^b^	45.14 ± 0.12^n^	35.00 ± 0.08^i^	1.37 ± 0.04^a^	1.07 ± 0.02^j^	2.51 ± 0.04^ef^	1.41 ± 0.03^efgh^	81.42 ± 0.19^no^	18.59 ± 0.19^ab^	4.38 ± 0.06^m^	1.29 ± 0.00^o^
P3	10.74 ± 0.06^de^	0.32 ± 0.04^ef^	3.32 ± 0.02^e^	45.28 ± 0.12^n^	34.60 ± 0.11^h^	1.41 ± 0.07^ab^	0.87 ± 0.02^fgh^	2.32 ± 0.01^bc^	1.16 ± 0.01^ab^	81.07 ± 0.18^n^	18.94 ± 0.18^b^	4.29 ± 0.05^l^	1.31 ± 0.00^p^
P4	11.82 ± 0.04^l^	0.24 ± 0.02^c^	3.83 ± 0.03^i^	39.80 ± 0.11^e^	37.48 ± 0.09^n^	1.61 ± 0.04^gh^	0.95 ± 0.01^i^	2.72 ± 0.05^gh^	1.57 ± 0.02^ij^	78.46 ± 0.18^cdef^	21.55 ± 0.18^jklm^	3.64 ± 0.04^bcde^	1.06 ± 0.00^f^
P5	11.32 ± 0.04^ij^	0.74 ± 0.01^k^	3.84 ± 0.02^i^	40.38 ± 0.11^f^	36.97 ± 0.08^m^	1.60 ± 0.01^fgh^	0.87 ± 0.01^gh^	2.94 ± 0.05^ij^	1.36 ± 0.03^defg^	78.95 ± 0.16^ghi^	21.05 ± 0.16^ghi^	3.76 ± 0.04^fgh^	1.09 ± 0.00^h^
P6	10.15 ± 0.05^a^	0.47 ± 0.01^h^	3.03 ± 0.03^c^	44.19 ± 0.11^l^	36.07 ± 0.08^k^	1.38 ± 0.01^ad^	0.97 ± 0.01^i^	2.42 ± 0.04^cde^	1.34 ± 0.05^cde^	81.70 ± 0.18^c^	18.31 ± 0.18^a^	4.47 ± 0.05^m^	1.23 ± 0.01^m^
P7	12.28 ± 0.07^o^	0.30 ± 0.01^de^	3.42 ± 0.03^fg^	38.12 ± 0.10^c^	39.80 ± 0.12^p^	1.47 ± 0.01^bc^	0.84 ± 0.04^efg^	2.45 ± 0.03^de^	1.33 ± 0.03^cde^	79.05 ± 0.17^hij^	20.95 ± 0.17^fgh^	3.78 ± 0.04^ghi^	0.96 ± 0.00^c^
P8	10.17 ± 0.06^a^	0.76 ± 0.04^k^	3.43 ± 0.04^fg^	46.10 ± 0.13^p^	33.92 ± 0.11^g^	1.42 ± 0.03^abc^	0.86 ± 0.03^fgh^	2.17 ± 0.02^a^	1.20 ± 0.01^ab^	81.63 ± 0.16^co^	18.38 ± 0.16^a^	4.45 ± 0.05^m^	1.36 ± 0.00^r^
P9	11.30 ± 0.06^hi^	0.36 ± 0.01^f^	4.21 ± 0.06^k^	40.67 ± 0.10^g^	37.55 ± 0.11^n^	1.64 ± 0.02^gh^	0.74 ± 0.01^abc^	2.36 ± 0.03 cd	1.19 ± 0.02^ab^	79.31 ± 0.18^ijko^	20.69 ± 0.18^efg^	3.83 ± 0.04^hi^	1.08 ± 0.00^g^
P10	11.47 ± 0.11^k^	0.35 ± 0.00^ef^	2.55 ± 0.06^a^	39.70 ± 0.13^e^	38.70 ± 0.12^o^	1.35 ± 0.01^a^	1.13 ± 0.02^k^	3.15 ± 0.01^kl^	1.63 ± 0.02^j^	79.87 ± 0.24^hm^	20.13 ± 0.24^c^	3.97 ± 0.06^k^	1.03 ± 0.00^e^
P11	10.84 ± 0.06^e^	0.26 ± 0.02 cd	4.13 ± 0.04^j^	36.78 ± 0.11^b^	40.85 ± 0.14^q^	1.72 ± 0.03^i^	0.84 ± 0.02^efg^	3.14 ± 0.05^k^	1.47 ± 0.03^hi^	78.72 ± 0.21^efg^	21.29 ± 0.21^hijk^	3.70 ± 0.04^defg^	0.90 ± 0.00^b^
P12	10.61 ± 0.06^c^	0.18 ± 0.00^ab^	3.12 ± 0.04^d^	36.10 ± 0.13^a^	40.86 ± 0.11^q^	1.51 ± 0.04^de^	1.47 ± 0.02^l^	4.17 ± 0.04^m^	2.00 ± 0.03^k^	78.60 ± 0.22^defg^	21.41 ± 0.22^ijkl^	3.68 ± 0.05^cdefg^	0.88 ± 0.00^a^
P13	10.45 ± 0.04^b^	0.42 ± 0.01^g^	5.08 ± 0.02^o^	39.84 ± 0.12^e^	36.58 ± 0.10^l^	2.08 ± 0.03^k^	0.83 ± 0.01^efg^	3.26 ± 0.03^l^	1.48 ± 0.07^hi^	77.66 ± 0.20^ab^	22.34 ± 0.20^no^	3.48 ± 0.04^a^	1.09 ± 0.00^h^
P14	11.18 ± 0.04^gh^	0.37 ± 0.01^f^	3.38 ± 0.03^ef^	44.69 ± 0.11^m^	33.91 ± 0.09^g^	1.50 ± 0.04^cde^	0.90 ± 0.04^h^	2.65 ± 0.08^g^	1.45 ± 0.08^fgh^	79.86 ± 0.18^m^	20.15 ± 0.18^c^	3.96 ± 0.05^k^	1.32 ± 0.00^q^
P15	11.32 ± 0.04^ij^	0.34 ± 0.02^ef^	3.70 ± 0.03^h^	47.29 ± 0.13^r^	31.35 ± 0.11^c^	1.49 ± 0.01^bcde^	0.83 ± 0.01^efg^	2.37 ± 0.10 cd	1.34 ± 0.02^c^	79.80 ± 0.20^lm^	20.20 ± 0.20 cd	3.95 ± 0.05^k^	1.51 ± 0.00^v^
P16	12.61 ± 0.08^p^	0.17 ± 0.02^a^	4.10 ± 0.03^j^	42.38 ± 0.11^i^	35.05 ± 0.13^i^	1.53 ± 0.03^pdef^	0.73 ± 0.02^ab^	2.21 ± 0.03^ab^	1.25 ± 0.02^bcde^	78.31 ± 0.20^de^	21.69 ± 0.20^klm^	3.61 ± 0.04^bcd^	1.21 ± 0.00^l^
P17	12.17 ± 0.02^no^	0.64 ± 0.06^j^	4.07 ± 0.03^j^	45.62 ± 0.11^o^	31.70 ± 0.08^d^	1.60 ± 0.03^fgh^	0.72 ± 0.01^ab^	2.36 ± 0.01 cd	1.14 ± 0.02^a^	78.67 ± 0.11^deh^	21.33 ± 0.11^hijk^	3.69 ± 0.03^cdefg^	1.44 ± 0.00 t
P18	11.91 ± 0.02^l^	0.25 ± 0.01^c^	3.87 ± 0.01^i^	40.80 ± 0.13^g^	36.40 ± 0.11^l^	1.64 ± 0.01^h^	0.80 ± 0.01^de^	3.00 ± 0.15^j^	1.34 ± 0.02^cde^	78.25 ± 0.22^d^	21.76 ± 0.22^lm^	3.60 ± 0.05^bc^	1.12 ± 0.00^i^
P19	11.44 ± 0.02^jk^	0.35 ± 0.02^ef^	3.71 ± 0.03^h^	45.94 ± 0.10^p^	32.87 ± 0.13^e^	1.49 ± 0.04^bcde^	0.82 ± 0.04^efg^	2.16 ± 0.03^a^	1.24 ± 0.04^bc^	79.97 ± 0.16^m^	20.03 ± 0.16^c^	4.00 ± 0.04^k^	1.40 ± 0.00^s^
P20	11.52 ± 0.05^k^	0.73 ± 0.01^k^	4.07 ± 0.03^j^	42.76 ± 0.12^j^	33.68 ± 0.10^f^	1.73 ± 0.04^i^	0.87 ± 0.01^gh^	3.27 ± 0.04^l^	1.39 ± 0.04^efgh^	78.04 ± 0.20^bc^	21.96 ± 0.20^mn^	3.55 ± 0.04^ab^	1.27 ± 0.00^n^
P21	11.02 ± 0.01^f^	0.55 ± 0.02^i^	3.49 ± 0.02^g^	47.66 ± 0.13^s^	30.53 ± 0.11^a^	1.56 ± 0.03^efg^	0.98 ± 0.02^i^	2.77 ± 0.08^h^	1.46 ± 0.04^gh^	79.71 ± 0.19^klm^	20.30 ± 0.19^cde^	3.93 ± 0.05^jk^	1.56 ± 0.00^w^
P22	11.51 ± 0.05^k^	0.73 ± 0.04^k^	4.26 ± 0.03^kl^	45.98 ± 0.13^p^	30.86 ± 0.10^b^	1.74 ± 0.04^i^	0.78 ± 0.01^cde^	2.90 ± 0.03^ij^	1.26 ± 0.04^bcd^	78.35 ± 0.19^cde^	21.66 ± 0.19^klm^	3.62 ± 0.04^bcd^	1.49 ± 0.00 u
P23	11.16 ± 0.01^g^	0.45 ± 0.01^gh^	4.28 ± 0.01^l^	41.26 ± 0.07^h^	36.03 ± 0.11^k^	1.77 ± 0.08^i^	0.86 ± 0.04^fgh^	2.90 ± 0.03^ij^	1.31 ± 0.00^cde^	78.59 ± 0.14^de^	21.41 ± 0.14^ijkl^	3.67 ± 0.03^cdef^	1.15 ± 0.01^k^
P24	10.72 ± 0.02^cde^	0.37 ± 0.01^f^	5.16 ± 0.02^p^	43.35 ± 0.09^k^	34.10 ± 0.12^g^	1.88 ± 0.01^j^	0.71 ± 0.03^a^	2.47 ± 0.10^de^	1.26 ± 0.01^bcd^	78.52 ± 0.16^defg^	21.49 ± 0.16^ijkl^	3.66 ± 0.04^cdef^	1.27 ± 0.00^n^
P25	12.11 ± 0.02^mn^	0.36 ± 0.01^f^	4.62 ± 0.04^n^	40.67 ± 0.10^g^	35.81 ± 0.09^j^	1.73 ± 0.02^i^	0.77 ± 0.01^bcd^	2.61 ± 0.07^fg^	1.35 ± 0.02^cdef^	77.60 ± 0.17^a^	22.40 ± 0.17^o^	3.47 ± 0.04^a^	1.14 ± 0.00^j^
P26	10.62 ± 0.04 cd	0.18 ± 0.01^ab^	4.54 ± 0.01^m^	46.61 ± 0.06^q^	31.27 ± 0.10^c^	1.85 ± 0.02^j^	0.82 ± 0.02^def^	2.82 ± 0.04^hi^	1.31 ± 0.03^cde^	78.87 ± 0.14^fgh^	21.13 ± 0.14^hij^	3.73 ± 0.03^efg^	1.49 ± 0.00 u

Average value													
*N*	11.10 ± 0.72^a^	0.37 ± 0.19^a^	3.42 ± 0.51^a^	40.91 ± 3.45^a^	37.62 ± 2.41^a^	1.49 ± 0.12^a^	0.95 ± 0.20^a^	2.73 ± 0.56^a^	1.41 ± 0.23^a^	79.85 ± 1.25^a^	20.15 ± 1.25^a^	3.98 ± 0.32^a^	1.10 ± 0.16^a^
S	11.41 ± 0.62^a^	0.42 ± 0.18^a^	4.16 ± 0.54^b^	43.92 ± 2.67^b^	33.58 ± 2.17^b^	1.68 ± 0.17^b^	0.81 ± 0.07^b^	2.70 ± 0.36^a^	1.32 ± 0.10^a^	78.73 ± 0.81^b^	21.27 ± 0.81^b^	3.71 ± 0.18^b^	1.32 ± 0.16^b^

Note

*N* means peanuts grown in the north; S means peanuts grown in the south; values in the columns with different letters (a‐w) are significantly different (*p* <.05).

Abbreviations: CPO, cold‐pressed peanut oil; SFAs, saturated fatty acids; UFA, unsaturated fatty acids; UFA/SFA, unsaturated to saturated fatty acids; O/L, oleic/linoleic ratio.

The appropriate proportion pattern of UFA/SFA has a positive role to guarantee the oxidation stability of vegetable oil and reduce the incidence of coronary heart disease (CHD). Either peanuts or processed peanuts have been confirmed to be beneficial for health mainly because of their desirable lipid profile, which is higher in unsaturated fatty acids than in saturated fatty acids (Akhtar et al., 2014). As described in Table 2, the percentage of unsaturated fatty acids (UFA), saturated fatty acids (SFA), and the ratio of UFA/SFA in examined samples were calculated. It was observed that CPOs possessed high levels of unsaturated fatty acids with the average value of 79.24% and less saturated fatty acids for 20.76%, which suggest that peanut oils could exert antioxidant properties, lower cholesterol, and even reduce the heart disease risk (Wang, 2018). The variation range of UFA/SFA value of peanut oils was 3.47–4.47, which was overlaid by equivalent data (2.05–4.62) collected from previous 45 samples analyzed by (Wang, 2018); the varieties with higher ratio were Shanhua9 (P6, 4.47), Luhua11(P8, 4.45), and Jihua4 (P2, 4.38).

As oleic and linoleic acid are so prevalent in peanut, the typical convention for reporting their concentrations is O/L ratio. High O/L characteristic could confer an evident health advantage to the consumer and has the potential to greatly enhance the marketability of peanuts. Cicero et al. (2008) has pointed there was an inverse relation between the O/L ratio of plasma LDL and biomarkers of oxidative stress according to a clinical trial. Moreover, O/L ratio is an important factor to estimate the stability of peanut oil and other derived products (Andersen & Gorbet, 2002; Young et al., 1974). A longer shelf life is relevant to higher ratio (Branch et al., 1990), which essentially attributed to that the linoleic acid with two double bonds is more susceptible to oxidative rancidity than oleic acid (one double bond)(Kratz et al., 2002). As observed in Table 2, remarkable differences were found within O/L ratios among varieties of peanut, and the range was 0.88–1.56, all lower than those (1.8–2.1) in literature (Mora‐Escobedo et al., 2014), which had a relationship with growth environment, cultivar, or processing methods (Grosso & Guzman, 1995). CPO from Tianfu18 (P21) grown in Sichuan contained higher oleic acid content and O/L ratio than the other varieties, suggesting its better oxidation resistance.

### 3.3 Bioactive components

Vegetable oil is one of the richest sources for vitamin E, which is as well as established to contribute to restrain oil deterioration. Accordingly, it is necessary to assess the oil tocopherol between different types. As shown in Table 3, CPOs had plentiful *α‐*, *γ‐,* and *δ*‐tocopherol, accounting for 61.98%, 36.03%, and 1.99% of the total and no tocotrienols could be detected. Among peanut accessions exanimated, the total tocopherol content showed significant difference (*p <*.01), which was in the range of 292–547 mg/kg and the mean was 390.65 mg/kg, accompanied with the highest amount for Silihong (P12) while Qinghua7 (P9) was at the lowest tocopherol level. Zhu et al. (2015) stated that the content of total tocopherols in commercially pressed crude peanut oil was 367 mg/kg, which was within the data range measured in the present work, but it would be reduced by 10.35% after chemical refining. Evidence (Kamal‐Eldin & Appelqvist, 1996) already proved the vitamin E distribution ratio in peanut oil was conducive to its function in the body, which was ascribed to the order of physiological activity of vitamin E isomers in the organism (*δ*‐tocopherol<γ‐tocopherol<α‐tocopherol). The *α*‐tocopherol, biologically and chemically the most active form of vitamin E, could act as the dominant lipid‐soluble antioxidant through breaking the lipid oxidation chain (Ekanayake‐Mudiyanselage et al., 2005). So Zhonghua21 (P25) from Hubei province with *α*‐tocopherol contents of 314.73 mg/kg could be inferred as the varieties with physiological activity and high antioxidant ability.

**TABLE 3:** Total tocopherols and total phytosterols, *β*‐sitosterol, stigmasterol, campesterol contents of CPOs from twenty‐six peanut cultivars grown in China (mg/kg)

Sample	*β*‐sitosterol	Stigmasterol	Campesterol	Total phytosterols	*α*‐ tocopherol	*γ*‐tocopherol	*δ*‐tocopherol	Total tocopherols
P1	843.43 ± 25.28^l^	197.59 ± 9.96^abcde^	136.44 ± 8.03^efg^	1177.46 ± 27.20^k^	282.46 ± 1.94^m^	104.45 ± 1.47^c^	9.91 ± 0.25^i^	396.82 ± 0.74^hi^
P2	1100.91 ± 50.27^n^	167.49 ± 6.98^a^	176.35 ± 8.19^hi^	1444.74 ± 65.44^lm^	247.71 ± 2.26^i^	136.59 ± 0.36^g^	7.59 ± 0.40^g^	391.88 ± 2.29^g^
P3	752.80 ± 16.36^k^	332.08 ± 14.88^j^	157.89 ± 10.83^gh^	1242.76 ± 42.07^k^	160.70 ± 0.11^a^	146.99 ± 1.20^h^	6.30 ± 0.32^def^	313.98 ± 0.77^bc^
P4	1054.54 ± 58.02^n^	277.72 ± 10.12^hi^	192.07 ± 9.22^ij^	1524.32 ± 38.68^m^	266.62 ± 1.61^l^	135.99 ± 0.76^fg^	14.17 ± 0.48^k^	416.76 ± 2.84^j^
P5	899.11 ± 33.63^lm^	302.65 ± 17.57^ij^	183.69 ± 10.08^ij^	1385.45 ± 61.28^l^	233.14 ± 0.69^g^	169.18 ± 3.22^jk^	4.41 ± 0.02^a^	406.72 ± 3.94^j^
P6	739.97 ± 21.79^jk^	253.84 ± 12.18^gh^	201.37 ± 14.97^j^	1195.18 ± 48.94^k^	266.68 ± 1.88^l^	133.96 ± 2.81^fg^	5.89 ± 0.20^cde^	406.51 ± 4.49^i^
P7	680.36 ± 28.15^ij^	171.82 ± 16.38^ab^	96.88 ± 10.35^bcd^	949.05 ± 54.89^hij^	269.15 ± 1.30^l^	159.11 ± 4.30^i^	9.73 ± 0.39^i^	437.99 ± 5.21^l^
P8	555.42 ± 27.90^efg^	193.82 ± 13.09^abcd^	96.02 ± 9.29^bcd^	845.25 ± 50.28^efgh^	242.88 ± 1.52^h^	138.12 ± 2.52^g^	8.16 ± 0.15^gh^	389.15 ± 0.85^g^
P9	404.66 ± 11.47^bc^	227.02 ± 16.91^defg^	94.31 ± 6.63^bc^	725.98 ± 35.00 cd	159.25 ± 6.63^a^	128.69 ± 1.99^e^	4.07 ± 0.21^a^	292.00 ± 8.83^a^
P10	910.58 ± 34.04^m^	303.19 ± 19.13^ij^	172.55 ± 11.31^hi^	1386.32 ± 3.60^l^	185.39 ± 0.18^b^	237.03 ± 4.51^m^	5.65 ± 0.07 cd	428.07 ± 4.26^k^
P11	662.51 ± 17.36^hi^	189.55 ± 5.65^abc^	131.97 ± 8.12^ef^	984.02 ± 31.13^ij^	256.77 ± 0.04^kj^	134.78 ± 1.47^fg^	4.40 ± 0.12^a^	395.94 ± 1.31^gh^
P12	1053.08 ± 50.97^n^	255.78 ± 9.26^gh^	313.54 ± 11.28^k^	1622.39 ± 30.43^n^	293.04 ± 0.67^n^	249.27 ± 2.53^n^	4.69 ± 0.54^ab^	547.00 ± 3.74^n^
P13	616.03 ± 20.46^gh^	185.57 ± 8.49^abc^	131.25 ± 9.13^e^	932.84 ± 38.08^ghi^	258.02 ± 2.96^jk^	145.05 ± 0.33^h^	13.00 ± 0.31^m^	416.07 ± 2.32^j^
P14	375.14 ± 28.79^ab^	312.61 ± 17.66^j^	65.24 ± 6.48^a^	752.98 ± 52.93^cde^	249.00 ± 0.64^i^	163.66 ± 3.22^ijk^	6.16 ± 0.49^def^	418.81 ± 2.10^j^
P15	367.00 ± 16.99^ab^	174.75 ± 12.67^ab^	77.73 ± 7.18^ab^	619.47 ± 36.84^ab^	226.74 ± 0.76^ef^	112.97 ± 4.84^d^	6.39 ± 0.41^def^	346.09 ± 3.67^d^
P16	400.70 ± 18.73^b^	224.81 ± 11.77^defg^	87.50 ± 3.54^b^	713.00 ± 34.04^bc^	254.97 ± 1.07^j^	108.05 ± 3.87 cd	6.79 ± 0.09^f^	369.80 ± 2.71^f^
P17	407.39 ± 15.80^bc^	178.83 ± 13.94^ab^	78.42 ± 4.19^ab^	664.63 ± 33.93^abc^	213.25 ± 0.57^d^	89.99 ± 2.47^b^	8.77 ± 0.61^h^	312.00 ± 2.42^b^
P18	567.22 ± 22.95^fg^	217.70 ± 9.96^cdef^	120.81 ± 7.29^ef^	905.72 ± 40.21^fghi^	260.07 ± 1.56^k^	88.98 ± 3.60^b^	6.78 ± 0.09^f^	355.82 ± 5.06^e^
P19	319.56 ± 14.16^a^	194.28 ± 12.32^abcd^	63.76 ± 5.49^a^	577.59 ± 31.98^a^	233.32 ± 1.89^g^	74.07 ± 0.25^a^	8.44 ± 0.18^h^	315.82 ± 1.81^bc^
P20	500.32 ± 20.73^de^	201.40 ± 15.49^abcde^	120.81 ± 8.83^ef^	822.52 ± 45.04^def^	226.20 ± 0.29^ef^	189.01 ± 1.82^l^	4.25 ± 0.07^a^	419.46 ± 2.03^j^
P21	694.23 ± 33.47^ijk^	228.70 ± 20.46^efg^	115.48 ± 14.31^cde^	1038.41 ± 68.25^j^	227.32 ± 3.26^f^	165.43 ± 6.09^jk^	9.99 ± 0.16^i^	402.73 ± 9.19^hi^
P22	467.51 ± 23.02 cd	245.36 ± 19.52^fg^	116.61 ± 8.55^de^	829.48 ± 51.09^efg^	299.99 ± 2.59^o^	148.14 ± 5.56^hi^	8.37 ± 0.30^h^	456.50 ± 7.86^m^
P23	652.96 ± 31.35^hi^	205.93 ± 21.73^bcde^	120.66 ± 14.43^ef^	979.54 ± 67.51^ij^	229.52 ± 2.88^fg^	158.02 ± 4.49^i^	5.17 ± 0.18^bc^	392.72 ± 1.79^g^
P24	519.29 ± 25.07^def^	223.60 ± 11.24^dfg^	138.40 ± 12.65^efg^	881.29 ± 48.97^fghi^	192.94 ± 1.27^c^	147.40 ± 1.63^h^	6.71 ± 0.53^f^	347.04 ± 0.17^d^
P25	561.14 ± 21.58^efg^	201.10 ± 15.52^abcde^	143.19 ± 10.94^fg^	905.42 ± 48.04^fghi^	314.73 ± 1.20^p^	124.39 ± 3.39^e^	19.07 ± 0.14^l^	458.19 ± 4.45^m^
P26	430.86 ± 19.64^bc^	377.23 ± 11.96^k^	87.54 ± 9.87^b^	895.63 ± 41.47^fghi^	222.33 ± 1.13^e^	93.84 ± 2.82^b^	6.57 ± 0.70^ef^	322.73 ± 2.39^c^

Average value								
*N*	804.78 ± 61.44^a^	239.38 ± 16.24^a^	162.76 ± 17.61^a^	1206.91 ± 81.29^a^	238.65 ± 13.22^a^	156.18 ± 12.60^a^	7.08 ± 0.87^a^	401.90 ± 18.14^a^
S	491.38 ± 30.77^b^	226.56 ± 14.81^a^	104.81 ± 7.26^b^	822.75 ± 36.99^b^	243.45 ± 8.76^a^	129.21 ± 9.38^a^	8.31 ± 1.01^a^	380.98 ± 13.25^a^

Note

*N* means peanuts grown in the north; S means peanuts grown in the south; values in the columns with different letters (a‐p) are significantly different (*p* <.05).

Considerable variability in phytosterols was detected among the peanut oils selected across the country (*p* <.01), the content varied between 577.59 mg/kg and 1622.39 mg/kg. The top three sterol‐containing CPOs were Silihong (P12, 1622.39 mg/kg), Yuhua10 (P4, 1524.32 mg/kg), and Jihua4 (P2, 1444.74 mg/kg). Phytosterols are not only known as an important antioxidant that protects the oil against autoxidation (Khallouki et al., 2003), but also have physiological properties such as anticancer (Awad et al., 2000), anti‐inflammatory, and antitumor (Mariod et al., 2011), with an ability to even lower plasma cholesterol and LDL cholesterol (Oliveira Godoy Ilha et al., 2020). Most plant species, such as peanuts, comprise dominant *β*‐sitosterol, which is one of the most representative phytosterol along with stigmasterol and campesterol present in concentrations of 319.56–1100.91 mg/kg, 167.49–377.23 mg/kg, and 63.76–313.54 mg/kg. In the present work, the *β*‐sitosterol content corresponds with the results obtained by Zhang et al. (2012) (388–1157 mg/kg), with an increase in the stigmasterol content than that reported in the same work (3.1–226 mg/kg); the latter may be influenced by genotype and growing environment.

### 3.4 Stability of peanut oil

The Rancimat method can automatically detect the change in the conductivity of water caused by the volatile substances, which is produced by oil oxidation such as carboxylic acids and draws a curve of the change in conductivity over time to obtain IP value under conditions of forced oxidation. As presented in Table 4, the antioxidant times of peanut oils could last for 6.98–10.61 hr. Antioxidant capacity may be associated with the oleic/linoleic ratio, sterols, and tocopherols. The correlation analysis revealed there was a positive correlation between the IP and oleic/linoleic ratio (*r* = 0.654, *p* <.01). Meanwhile, the IP showed no correlation with tocopherol and phytosterol.

**TABLE 4:** IP value (h) of CPOs from twenty‐six peanut cultivars grown in China

Sample	IP	Sample	IP	Sample	IP	Sample	IP
P1	7.29 ± 0.20^abc^	P8	9.16 ± 0.21^k^	P15	8.69 ± 0.10^hij^	P22	10.61 ± 0.25^l^
P2	8.61 ± 0.23^hi^	P9	7.17 ± 0.11^ab^	P16	8.15 ± 0.13^efg^	P23	8.25 ± 0.11^fgh^
P3	8.06 ± 0.20^ef^	P10	8.08 ± 0.11^ef^	P17	8.57 ± 0.11^hi^	P24	8.37 ± 0.14^fghi^
P4	7.32 ± 0.14^abc^	P11	6.98 ± 0.14^a^	P18	7.84 ± 0.10^de^	P25	9.00 ± 0.11^jk^
P5	7.35 ± 0.20^bc^	P12	7.33 ± 0.17^abc^	P19	8.47 ± 0.13^ghi^	P26	9.15 ± 0.08^k^
P6	8.45 ± 0.17^ghi^	P13	7.65 ± 0.11 cd	P20	7.42 ± 0.23^bc^		
P7	8.29 ± 0.14^fgh^	P14	8.28 ± 0.13^fgh^	P21	7.87 ± 0.08^de^		

Average value							
*N*	7.84 ± 0.69^a^	S	8.45 ± 0.79^b^				

Note

*N* means peanuts grown in the north; S means peanuts grown in the south; values in the columns with different letters (a‐l) are significantly different (*p* <.05).

Abbreviations: CPO, cold‐pressed peanut oil; IP, oxidation induction period.

This situation was most likely to related to the high level of unsaturated fatty acids in peanut oil (>70%), while the low contents of endogenous vitamin E, phytosterols, and other antioxidant substances were not effective enough in preventing the oxidation of peanut oil. Therefore, it was reasonable to consider that peanut oil was easily oxidized in the storage and edible procedure, and the generated peroxide was quickly decomposed into aldehydes and ketones, which caused the rancidity of peanut oil (Balasubramaniam et al., 2019) and reduced the quality of peanut oil.

### 3.5 Effect of the growing regional on peanut oil

The north and south regions, as China's most important geographical division, may affect peanut varieties and their oil products’ quality. Considering the practical production demand, it is critical to master the qualification difference of CPOs in different planting sites of China. In this study, main quality indexes of peanut oils from two major growing areas were analyzed as follows. Taking oil content into consideration first, the mean value of the northern samples was 50.20%, while the slightly lower value of 49.28% appeared in the south, and significant differences were not found (*p* >.05). This finding coincided with the conclusion drawn by Zhang et al. (2006) that the oil content of rapeseed increases with the elevation of latitude in the planting area. The physicochemical properties of CPO illustrated acid values of northern samples varied from 0.11 mg KOH/g to 0.32 mg KOH/g, which were far lower than the values for southern oils (0.13–2.74 mg KOH/g); moreover, PVs of oils were 1.94–5.55 mmol/kg with a mean of 3.11 mmol/kg for northern peanuts and 2.04–4.91 mmol/kg with a mean of 3.12 mmol/kg for southern peanuts, respectively. It could be seen from the analysis that the above two indicators did not show a significant difference in value (*p >*.05). As far as fatty acids, no significant difference appeared in palmitic acid (C16:0) between CPOs from two regions (*p >*.05) with the average percentage of 11.10% and 11.41%. As a monounsaturated omega‐9 group fatty acid, oleic acid (C18:1) provides hypolipidemic effects and can prevent cardiovascular diseases (Jones et al., 2014). Here, the oleic acid of northern CPOs was measured to be 40.91%, was significantly lower than that of southern varieties (43.92%), which suggested that peanuts in southern China were more beneficial for human nutrition than northern samples as a whole owing to its higher content of oleic acid (Matthaus & Musazcan Ozcan, 2015). In addition, linoleic acid is omega‐6 group fatty acids, which in the north with the average content of 37.62% were found to be conspicuously higher than that (33.58%) of the other region. The increase in linoleic acid accompanying a decrease in oleic acid appears to be more marked in the south varieties and the largest change in composition appears to be in the Tianfu18 variety. Regarding the UFA/SFA ratio, higher value was obtained from north in both regions. This trend was similar to the conclusion published by Caporaso (2016), who suggested the ratio of unsaturated fatty acids (UFA) to saturated fatty acids (SFA) increased with the increase of latitude. However, the southern samples exhibited greater O/L ratio (1.32) in comparison to northern samples (1.10) with a significant difference (*p <*.01). Of the twenty‐six varieties examined, Chinese peanuts showed significant differences in phytosterols relative to planting sites, where northern content ranged from 725.98 mg/kg to 1622.39 mg/kg with an average of 1206.91 mg/kg, and southern content ranged from 577.59 mg/kg to 1038.41 mg/kg with an average of 822.75 mg/kg; *γ*‐tocopherol and total tocopherol at a higher amount with 156.18 mg/kg and 401.90 mg/kg in the CPOs originated from the north when compared to southern samples. It could be seen from the induction time analysis that southern samples had a mean IP of 8.45 hr, 7.78% higher than that of northern samples with significant differences (*p* <.05), which indicated that peanut oil obtained from southern China might be relatively more stable as a whole.

### 3.6 Comprehensive score and cluster analysis

Principal component analysis (PCA) was performed to reveal the relationships among twenty‐six CPOs from eleven provinces in China based on the nutrition component (phytosterol composition, tocopherol composition, oleic acid, linoleic acid, and IP) under consideration. In order to avoid the impact of the dimension and order of magnitude of each indicator on the evaluation result of peanut oil, the original data were standardized. The result showed that the cumulative variance contribution rate for the first three principal components was 77.01% (PC1=41.63%, PC2=20.64%, and PC3=14.74%, respectively, Table 5). PC1 mainly included the campesterol, *β*‐sitosterol, *γ*‐tocopherol, and linoleic acid content (principal component loading was 0.43, 0.42, 0.34, and 0.46, respectively), PC2 mainly included the *δ*‐tocopherol and *α*‐tocopherol content (principal component loading was 0.57 and 0.59, respectively), whereas PC3 was contributed by the stigmasterol content (0.41) and IP value (0.49). Consequently, the three principal components instead of the original 9 indicators could evaluate the CPOs’ nutritional quality.

**TABLE 5:** Variance contribution ratios of principal components of the quality characteristics of CPOs from twenty‐six peanut cultivars grown in China

Component	Initial eigenvalue			Extraction Sums of Squared Loadings		
	Total	% of Variance	Cumulative %	Total	% of Variance	Cumulative %
1	3.75	41.63	41.63	3.75	41.63	41.63
2	1.86	20.64	62.27	1.86	20.64	62.27
3	1.33	14.74	77.01	1.33	14.74	77.01
4	0.67	7.48	84.49			
5	0.56	6.22	90.70			
6	0.42	4.63	95.33			
7	0.25	2.78	98.10			
8	0.14	1.54	99.64			
9	0.03	0.37	100.00			

According to the eigenvalue in Table 5 and principal component loading, the function expressions of the three principal components were obtained:

$$Y_{1}=0.{\text{43X}}_{1}+0.0{\text{4X}}_{2}+0.{\text{42X}}_{3}‐0.0{\text{5X}}_{4}+0.{\text{34X}}_{5}+0.{\text{11X}}_{6}‐0.{\text{44X}}_{7}+0.{\text{46X}}_{8}‐0.{\text{34X}}_{9}$$

$$Y_{2}=‐0.0{\text{4X}}_{1}‐0.{\text{45X}}_{2}+0.0{\text{2X}}_{3}+0.{\text{57X}}_{4}‐0.{\text{25X}}_{5}+0.{\text{59X}}_{6}‐0.{\text{17X}}_{7}+0.{\text{11X}}_{8}+0.{\text{14X}}_{9}$$

$$Y_{3}=0.{\text{37X}}_{1}+0.{\text{41X}}_{2}+0.30X_{3}+0.{\text{21X}}_{4}+0.{\text{29X}}_{5}+0.{\text{27X}}_{6}+0.{\text{28X}}_{7}‐0.{\text{28X}}_{8}+0.{\text{49X}}_{9}$$

X_1_‐campesterol, X_2_‐stigmasterol, X_3_‐*β*‐sitosterol, X_4_‐*δ*‐tocopherol, X_5_‐*γ*‐tocopherol, X_6_‐*α*‐tocopherol, X_7_‐oleic acid, X_8_‐linoleic acid, X_9_‐IP.

Then, the proportion of eigenvalue corresponding to each principal component to the sum of the total eigenvalue of the extracted principal component was used as the weight to calculate the comprehensive model of the principal component:

$$Y\;=0.{\text{54Y}}_{1}+0.{\text{27Y}}_{2}+0.{\text{19Y}}_{3}$$

Finally, according to the comprehensive evaluation function, the comprehensive scores of twenty‐six CPOs were calculated. The higher the comprehensive score, the better the nutritional quality of the peanut oil. As it can be seen from Table 6, among the top 10 samples, there were seven peanut varieties from northern China, and Silihong (P12), Yuhua 10 (P4), and Huayu 18 (P1) were the top three regions. The highest comprehensive score of peanut variety from southern China was Zhonghua 21 (P25) which was 1.08.

**TABLE 6:** Comprehensive evaluation of CPOs from twenty‐six peanut cultivars grown in China

Ranking	Code	Growing location	Score
NO.1	P12	Liaoning Province	3.05
NO.2	P4	Kaifeng City, Henan Province	1.65
NO.3	P1	Hengshui City, Hebei Province	1.17
NO.4	P25	Xiaogan City, Hubei Province	1.08
NO.5	P10	Xuzhou City, Jiangsu Province	0.88
NO.6	P7	Rushan City, Shandong Province	0.87
NO.7	P11	Jingzhou City, Liaoning Province	0.84
NO.8	P5	Puyang City, Henan Province	0.83
NO.9	P13	Huaian City, Jiangsu Province	0.68
NO.10	P2	Baoding City, Hebei Province	0.63
NO.11	P6	Linyi City, Shandong Province	0.47
NO.12	P23	Gucheng City, Hubei Province	−0.05
NO.13	P18	Guangxi Province	−0.07
NO.14	P20	Jiangxi Province	−0.40
NO.15	P22	Yongzhou City, Hunan Province	−0.55
NO.16	P21	Nanchong City, Sichuan Province	−0.59
NO.17	P3	Shijiazhuang City, Hebei Province	−0.60
NO.18	P16	Nanchang City, Jiangxi Province	−0.64
NO.19	P24	Xiangyang City, Hubei Province	−0.64
NO.20	P8	Jiaozhou City, Shandong Province	−0.68
NO.21	P14	Jiangxi Province	−0.92
NO.22	P9	Yantai City, Shandong Province	−0.96
NO.23	P17	Zhanjiang City, Guangdong Province	−1.43
NO.24	P19	Guangxi Province	−1.50
NO.25	P15	Ganzhou City, Jiangxi Province	−1.55
NO.26	P26	Huanggang City, Hubei Province	−1.58

Note

NO.1‐NO.26 represents the peanut oil ranking from high to low comprehensive score.

**FIGURE 1:**
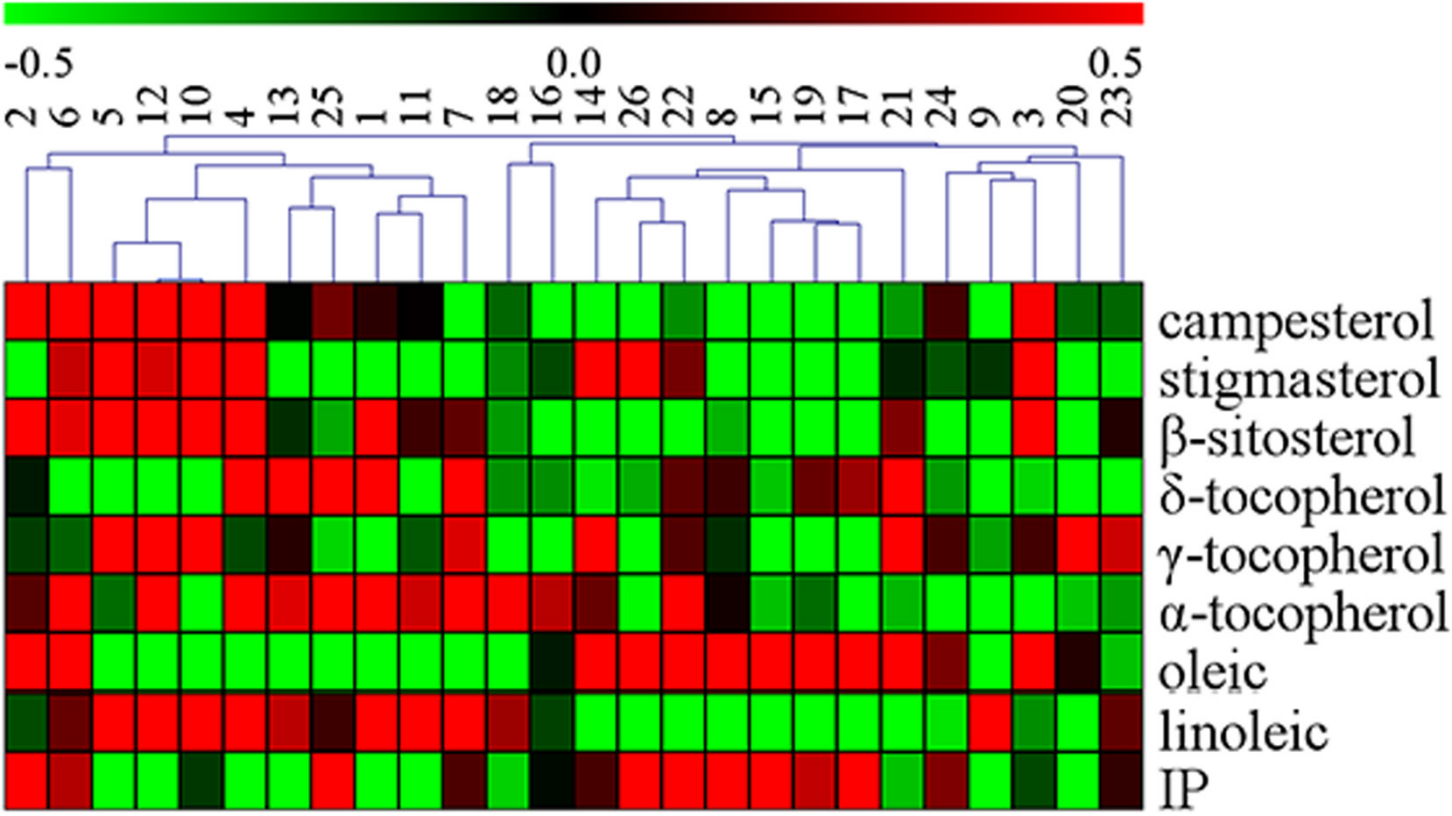
Heat map of all peanut seed oils using composition data for sterols, tocopherols, oleic acid, linoleic acid, and IP. The way cluster analysis indicates the closeness of the peanut species (horizontal) to each other. 1–26 represents peanut oils named P1–P26 in the paper

To further analyze the nutrition component of cold‐pressed peanut oil from different varieties, the results were presented as a heat map added to the dendrogram (Figure 1). As seen from the picture, twenty‐six samples were divided into two categories. P12, P4, P1, P25, P10, P7, P11, P5, P13, P2, P6 (No.1–11) were clustered together to form the first group, and their score was above 0. The heat map showed that the content of campesterol, *β*‐sitosterol, *α*‐tocopherol, and linoleic in the first group was higher than that of other group which is consistent with the result of comprehensive evaluation based on PCA. On the whole, it could be concluded that the top ranking of peanut oil quality is mostly in the north.

## 4 CONCLUSIONS

For the current study, peanut varieties in China contained relatively high levels of oil varied from 45.97% to 57.28% and rich in oleic (36.10%‐47.66%) and linoleic acid (30.53%‐40.86%). Meanwhile, CPOs could be considered a good nutrition source, which consist of copious *α*‐,*γ*‐, and *δ*‐tocopherol and *β*‐sitosterol, stigmasterol, campesterol. The physicochemical characteristics under evaluation were as follows: the acid value (0.11–2.74 mg KOH/g) and peroxide value (1.94–5.55 mmol/kg). Besides, CPOs had strong oxidation stability with an induction period of more than 7 hr. Among all tested varieties, Silihong could be characterized for exhibiting higher nutritional level with the comprehensive scores of 3.05, followed by Yuhua10 (1.65) and Huayu18 (1.17). The cluster analysis results indicated that most of the peanut samples with scores above 0 were from northern China. Conclusively, it was preliminarily believed that high‐quality cold‐pressed oil might be easy to form in peanut varieties from northern China, but further research is necessary.

## CONFLICT OF INTEREST

The authors declare that they have no conflict of interest.

## AUTHOR CONTRIBUTION

**Huang Ying:** Conceptualization (lead); Investigation (equal); Methodology (lead); Writing – original draft (lead). **Liu Changsheng:** Data curation (equal); Supervision (equal). **Huang Fenghong:** Funding acquisition (equal); Project administration (equal); Resources (equal); Writing – review & editing (equal). **Zhou Qi:** Formal analysis (supporting); Funding acquisition (supporting); Software (supporting). **chang zheng:** Data curation (equal); Formal analysis (lead); Software (equal); Writing – review & editing (lead). **Liu Rui:** Funding acquisition (supporting); Resources (supporting). **Huang Jiazhang:** Project administration (supporting); Supervision (supporting).

## Supporting information

Supplementary Material
