# Supplementary material for: Quality evaluation of oil by cold‐pressed peanut from different growing regions in China
Source: Food Sci Nutr. 2022 Mar 14;10(6):1975–87. doi: 10.1002/fsn3.2813 (PMC9179141; doi:10.1002/fsn3.2813)
Supplement: Supplementary file 1 — Supplementary Material [file FSN3-10-1975-s001.docx]

**TABLE S1**. Geographical sources and classification information of twenty-six peanut cultivars grown in China

| Code | Variety name | Growing location | Classification |
| --- | --- | --- | --- |
| P1 | Huayu18 | Hengshui City, Hebei Province | N |
| P2 | Jihua4 | Baoding City, Hebei Province | N |
| P3 | Luhua14 | Shijiazhuang City, Hebei Province | N |
| P4 | Yuhua10 | Kaifeng City, Henan Province | N |
| P5 | Huayu16 | Puyang City, Henan Province | N |
| P6 | Shanhua9 | Linyi City, Shandong Province | N |
| P7 | Haihua2 | Rushan County, Shandong Province | N |
| P8 | Luhua11 | Jiaozhou County, Shandong Province | N |
| P9 | Qinghua7 | Yantai City, Shandong Province | N |
| P10 | Luhua9 | Xuzhou City, Jiangsu Province | N |
| P11 | Local-Baisha | Jingzhou City, Liaoning Province | N |
| P12 | Silihong | Liaoning Province | N |
| P13 | Local red peanut | Huaian City, Jiangsu Province | S |
| P14 | Yueyou58 | Jiangxi Province | S |
| P15 | Shanyou21 | Ganzhou City, Jiangxi Province | S |
| P16 | Zhongkai Flower1 | Nanchang City, Jiangxi Province | S |
| P17 | Zhanyou75 | Zhanjiang City, Guangdong Province | S |
| P18 | Heyou12 | Guangxi Province | S |
| P19 | Guiyou28 | Guangxi Province | S |
| P20 | Little peanut with red skin | Yiyang City, Hunan Province | S |
| P21 | Tianfu18 | Nanchong City, Sichuan Province | S |
| P22 | Xiangnong small peanut | Yongzhou City, Hunan Province | S |
| P23 | Baisha1016 | Gucheng County, Hubei Province | S |
| P24 | Dabaisha | Xiangyang City, Hubei Province | S |
| P25 | Zhonghua21 | Xiaogan City, Hubei Province | S |
| P26 | Zhonghua 5 | Huanggang City, Hubei Province | S |

Note: N means peanuts grown in the North; S means peanuts grown in the South.
